# The complete plastid genome sequence of *Quercus acuta* (Fagaceae), an evergreen broad-leaved oak endemic to East Asia

**DOI:** 10.1080/23802359.2020.1866449

**Published:** 2021-02-03

**Authors:** Won-Bum Cho, Eun-Kyeong Han, In-Su Choi, Myounghai Kwak, Jung-Hyun Kim, Bo-Yun Kim, Jung-Hyun Lee

**Affiliations:** a Department of Biology Education, Chonnam National University, Gwangju, Republic of Korea; b Department of Biological Sciences and Biotechnology, Chonnam National University, Gwangju, Republic of Korea; c School of Life Sciences, Arizona State University, Tempe, AZ, USA; d Biological and Genetic Resources Utilization Division, National Institute of Biological Resources, Incheon, Republic of Korea; e Plant Resources Division, National Institute of Biological Resources, Incheon, Republic of Korea

**Keywords:** Complete plastid genome, *Cyclobalanopsis*, evergreen broad-leaved oak, phylogenetic analysis, *Quercus acuta*

## Abstract

We are reporting the complete plastid genome (plastome) of *Quercus acuta*, an evergreen broad-leaved oak endemic to East Asia. This species is important for maintaining the warm-temperate evergreen forest biome in East Asia. The *Q. acuta* plastome is 160,522 base pairs (bp) long, with two inverted repeat (IR) regions (25,839 bp each) that separate a large single copy (LSC) region (90,199 bp) and a small single copy (SSC) region (18,645 bp). The phylogenetic tree shows that *Quercus acuta* is closely related to *Quercus sichourensis* with strong bootstrap support.

The genus *Quercus* L. is one of the most abundant and economically important woody plant genera in the Northern Hemisphere (Manos et al. 1999). Its evolutionary success is attributed to the species diversity, which varies among geographic regions and follows ecological divergence within each region (Han et al. 2020; Hipp et al. 2020). *Quercus* species can be locally endemic or widespread, extending from the equator to the boreal regions of Europe and from sea level to 4000 m in China (Kremer and Hipp 2020). *Quercus* section *Cyclobalanopsis* species dominate in the subtropical and warm-temperate evergreen broad-leaved forests of East Asia (Deng et al. 2018) where species such as *Quercus acuta* Thunb. and *Quercus hondae* Makino maintain a high degree of endemism (Qian and Ricklefs 2000).

To date, many plastid genomes (plastomes) have been sequenced for *Quercus* and have provided useful phylogenetic information to solve the taxonomical problems attributed to hybridization (Yang et al. 2016). However, *Cyclobalanopsis* plastome sequence availability is scarce, especially when considering their high level of endemism in East Asia. *Quercus acuta* is known as an evergreen broad-leaved oak tree endemic to the warm-temperate regions of East Asia, such as China, Korea and Japan. This species is an important resource for maintaining regional forest biomes (Lee and Choi 2010). However, their distribution in China, along with taxonomic problems, still remains controversial (Ohashi et al. 2006; Deng et al. 2008). Therefore, the plastom sequence for this species will be an important basis for improving our understanding of the evolutionary process and solving taxonomic problems in inter/intra species within especially section *Cyclobalanopsis*.

*Quercus acuta* samples were collected from Jeju Island, South Korea (N33°19′11″, E126°37′25″). The voucher specimen (MFDS-C-7840) was stored in the herbarium at the Korea Institute of Oriental Medicine (KIOM: sgyang81@kiom.re.kr). The DNA library was constructed and sequenced on the MGI-seq 2000 platform (LAS, Seoul, Korea) and generated 71,993,390 raw reads (150 bp paired-end). The *Q. acuta* plastid genome was constructed using NOVOPlasty 4.1 (Dierckxsens et al. 2017), with the *Q. gilva rbc*L gene sequence (Zeng et al. 2019; MK986651) as the seed. The sequence was checked by reference mapping using Geneious 10.2.3 (Kearse et al. 2012), on which 86,571 reads were assembled with an 80X coverage. The annotation was separately performed in Geneious 10.2.3 (Kearse et al. 2012), and was manually corrected for the start and stop codons and for the intron/exon boundaries. The annotated plastome sequence was deposited in GenBank (accession number: MT742291). The phylogenetic tree was constructed by downloading the complete plastome sequences of 20 related species (*Quercus*: 19 species, *Lithocarpus*: 1 species) from the NCBI database and alignments were performed with MAFFT (Katoh and Toh 2010). The maximum likelihood (ML) analysis was performed with RAxML v.8.0 (Stamatakis 2014) using default parameters and 1000 bootstrap replicates. For RAxML tree, the general time-reversible (GTR) model of nucleotide substation was used with the Gamma model of rate heterogeneity.

The *Q. acuta* plastome is 160,522 bp long, with two inverted repeat (IR) regions (25,839 bp each) that separate a large single copy (LSC) region (90,199 bp) and a small single copy (SSC) region (18,645 bp). This is the second shortest reported *Quercus* plastome. It contains 130 genes that encode 85 proteins, eight ribosomal RNAs, and 37 transfer RNAs, and the G + C content is 36.9% overall, 34.8% in the LSC region, and 42.8% in the IR regions. The gene content and order were comparable to other published *Quercus* plastome (Yang et al. 2016; Zeng et al. 2019). The phylogenetic tree (Figure 1) shows that *Q. acuta* is closely related to *Q. sichourensis*, with strong bootstrap support. Section *Cyclobalanopsis*, comprised of *Q. acuta*, *Q. sichourensis*, *Q. glauca*, and *Q. obovatifolia,* form a monophyletic sister clade to *Q. spinosa*. These data are useful for the phylogenetic and evolutionary studies of *Quercus* and Fagaceae.

**Figure 1.**
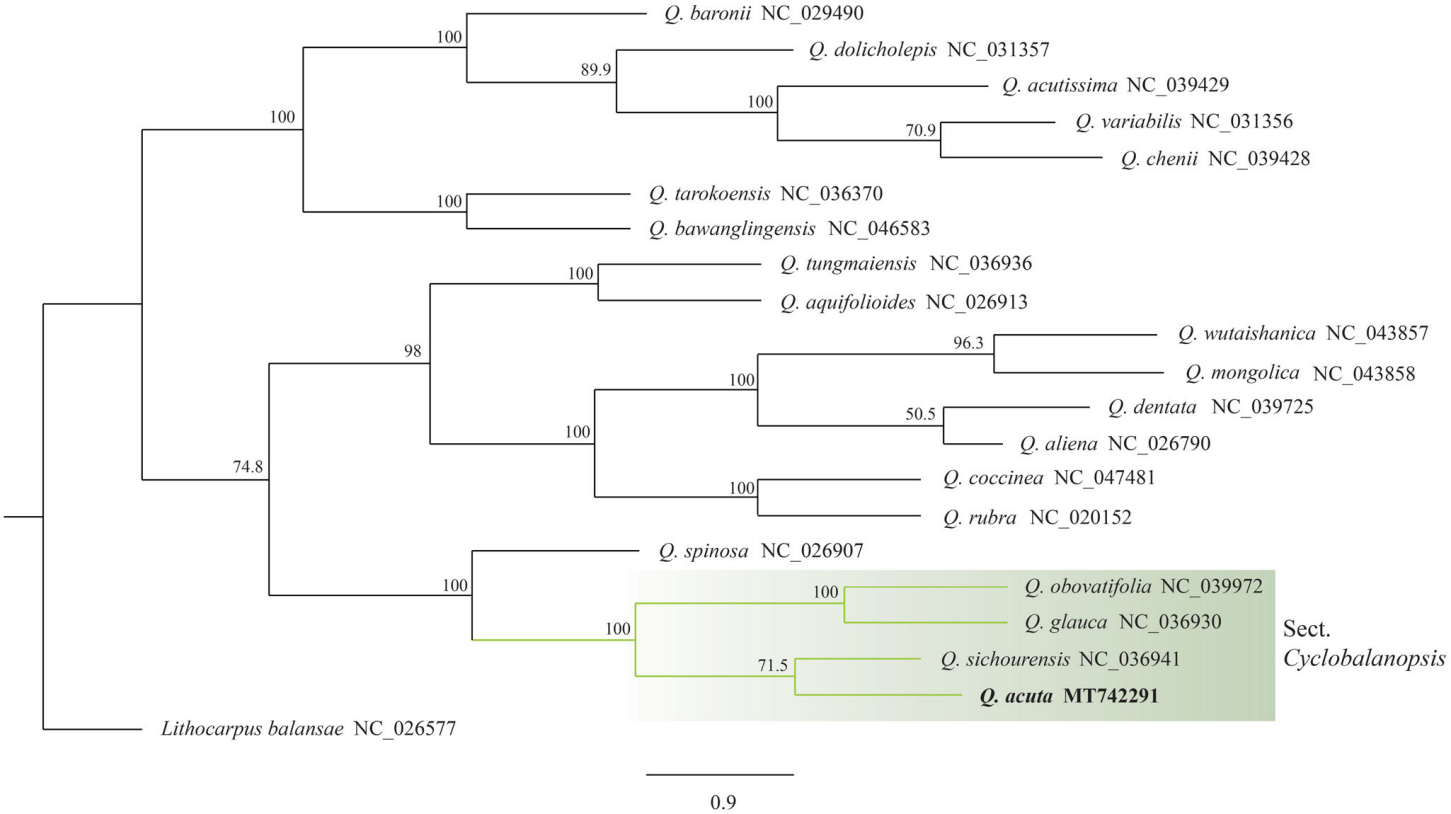
A phylogenetic tree (RAxML) using the plastome sequences of 21 species, with *Lithocarpus balansae* as the outgroup. The numbers above each node indicate the bootstrap value with 1000 replicates.

## Data Availability

The data that support the findings of this study are openly available in GenBank of NCBI (accession no. MT742291) at https://www.ncbi.nlm.nih.gov. All high-throughput sequencing data files are available from the GenBank Sequence Read Archive (SRA) accession number: SRR13068430.

## References

[CIT0001] Deng M, Cao LM, Cao M. 2008. A new distribution record of *Quercus acuta* Thunberg in Hongkong and Guangdong Province. Guihaia. 28:731–734.

[CIT0002] Deng M, Jiang XL, Hipp AL, Manos PS, Hahn M. 2018. Phylogeny and biogeography of East Asian evergreen oaks (*Quercus* section *Cyclobalanopsis*; Fagaceae): insights into the Cenozoic history of evergreen broad-leaved forests in subtropical Asia. Mol Phylogenet Evol. 119:170–181. 29175095 10.1016/j.ympev.2017.11.003

[CIT0003] Dierckxsens N, Mardulyn P, Smits G. 2017. NOVOPlasty: *de novo* assembly of organelle genomes from whole genome data. Nucleic Acids Res. 45(4):e18. 28204566 10.1093/nar/gkw955 PMC5389512

[CIT575023] Han EK, Cho WB, Park JS, Choi IS, Kwak M, Kim BY, Lee JH. 2020. A Disjunctive marginal edge of evergreen broad-Leaved oak (*Quercus gilva*) in East Asia: the high genetic distinctiveness and unusual diversity of Jeju Island populations and insight into a massive, independent postglacial colonization. Genes. 11(10):1114. 10.3390/genes11101114 PMC7598624 32977695

[CIT0004] Hipp AL, Manos PS, Hahn M, Avishai M, Bodénès C, Cavender ‐Bares J, Crowl AA, Deng M, Denk T, Fitz‐Gibbon S, et al. 2020. Genomic landscape of the global oak phylogeny. New Phytol. 226(4):1198–1212. 31609470 10.1111/nph.16162

[CIT0005] Katoh K, Toh H. 2010. Parallelization of the MAFFT multiple sequence alignment program. Bioinformatics. 26(15):1899–1900. 20427515 10.1093/bioinformatics/btq224 PMC2905546

[CIT0006] Kearse M, Moir R, Wilson A, Stones-Havas S, Cheung M, Sturrock S, Buxton S, Cooper A, Markowitz S, Duran C, et al. 2012. Geneious basic: an integrated and extendable desktop software platform for the organization and analysis of sequence data. Bioinformatics. 28(12):1647–1649. 22543367 10.1093/bioinformatics/bts199 PMC3371832

[CIT0007] Kremer A, Hipp AL. 2020. Oaks: an evolutionary success story. New Phytol. 226(4):987–1011. 31630400 10.1111/nph.16274 PMC7166131

[CIT0008] Lee JH, Choi BH. 2010. Distribution and northernmost limit on the Korean Peninsula of three evergreen trees. Korean J Pl Taxon. 40(4):267–273.

[CIT0009] Manos PS, Doyle JJ, Nixon KC. 1999. Phylogeny, biogeography, and processes of molecular differentiation in *Quercus* subgenus *Quercus* (Fagaceae). Mol Phylogenet Evol. 12(3):333–349. 10413627 10.1006/mpev.1999.0614

[CIT0010] Ohashi H, Ohashi K, Takahashi K. 2006. Identity of *Quercus acuta* Thunb. (Fagaceae) recorded from Taiwan and China. J Japanese Bot. 81:268–274.

[CIT0011] Qian H, Ricklefs RE. 2000. Large-scale processes and the Asian bias in species diversity of temperate plants. Nature. 407(6801):180–182. 11001054 10.1038/35025052

[CIT0012] Stamatakis A. 2014. RAxML version 8: a tool for phylogenetic analysis and post-analysis of large phylogenies. Bioinformatics. 30(9):1312–1313. 24451623 10.1093/bioinformatics/btu033 PMC3998144

[CIT0013] Yang Y, Zhou T, Duan D, Yang J, Feng L, Zhao G. 2016. Comparative analysis of the complete chloroplast genomes of five *Quercus* species. Front Plant Sci. 7:959. 27446185 10.3389/fpls.2016.00959 PMC4923075

[CIT0014] Zeng QM, Liu B, Lin RQ, Jiang YT, Liu ZJ, Chen SP. 2019. The complete chloroplast genome sequence of *Quercus gilva* (Fagaceae). Mitochondrial DNA Part B. 4(2):2493–2494. 33365597 10.1080/23802359.2019.1637299 PMC7687598

